# Practical Departments

**Published:** 1895-10-05

**Authors:** 


### PRACTICAL DEPARTMENTS.

### DISINFECTION AND THE EXISTING DATA FOR ITS PRACTICE.—I.

Disinfection in this country proceeds, to a large extent, by rule of thumb. While the etiology of infectious diseases had only a speculative basis, and no one was in a position to do more than guess at the action of any process or substance in disinfection, no other practice was possible. Of late years, however, more exact data have become available; the investigations which have furnished them have been various, both in their subject matter and in their methods; they are separated not only by vast masses of more attractive research in the journals which have published them, but still more by the diversity of method employed and to some extent of conclusions obtained. In the press of daily work it is not to be expected that medical officers of health and borough surveyors should be familiar with the papers in which these results are buried, and still less that they should undertake the minute examination which is necessary to obtain from such data a coherent and consistent answer to the problems which in practice have to be solved.

We therefore welcome a work by Dr. Rideal which will to some extent supply this want; and a careful perusal of it only emphasises the need which exists for such a manual.* The book is entitled, "An Introduction." The proper and, indeed, the only scientific "introduction" to the study of the subject would consist in an accurate and comparative investigation of a limited number of methods. Such study would teach not only exact data on the principal fundamental points, but also the methods of criticism which are necessary to obtain a conspective view from the consideration of conflicting results. This would serve as a guide for the reader when he has to undertake similar investigations on his own account. In the present volume too much attention is given to a consideration of a very large number of, sometimes, quite immaterial alternatives, and very rarely indeed is any single process examined in such detail as to leave the reader master of it at the finish. The mass of such alternatives is far greater than any person requiring an introduction could possibly assimilate, and, in regard to the large majority of them, the information given is of a superficial character. Had the volume been intended as a mere index or *catalogue raisonné* for what has been done on the subject, its title might have been ill-chosen, but it would, nevertheless, have served a useful end. Great, however, as is the variety of means alluded to, it is very far from exhausting those which have in practice been proposed.

This work, accordingly, somewhat fails in its object; but it would be extremely unfair to leave the impression that the book contains nothing worthy of the reputation of its author as a scientific chemist. On the subjects which it first treats, those of disinfection by light and by mechanical means, the treatment, though short, is perfectly adequate. In regard to the mechanical sterilisation of water there is no hesitation in rejecting the ordinary methods of filtration in favour of that proposed by Pasteur and Chamberland. Dr. Rideal refers to the possibility that a similar result might be obtained from the Berkefeld infusorial earth imitation; but, as the experiments of Dr. Johnston in the Public Health Laboratory of the Edinburgh University have shown, there is a wide difference between the efficiency of the two, and Dr. Rideal states, very accurately, in a later part of his book, that absolute safety in drinking water can at present only be obtained by boiling, or by the use of a Pasteur filter. The security which can be gained in this way is well shown in the various reports of the French War Office on the results obtained by the use of the Pasteur filter. The earlier reports of M. de Freycinet showed a continuous decrease in enteric fever, the disease with which the authorities were most harassed, as the use of the filters extended; and his last report in 1892 stated that enteric fever had disappeared wherever these filters had been applied. The recent report of General Zurlinden arrives at the same conclusion, and shows by a dissection of the results that this reduction was independent of the quality of the unfiltered water. Garrisons using the filters remained unattacked by enteric fever and cholera, even when these diseases were epidemic or endemic in the surrounding population using the same water; the removal or disuse of the filters was followed by outbreak of the disease. At the recent London Congress the British Medical Association paid a visit to an installation of Pasteur filters which Messrs. Debenham and Freebody have provided for their staff, roughly 1,000 in number; and the ease with which a copious supply of sterilised water was obtained, both in those parts of the premises where pressure was available and those where it was not, disposed of the impression sometimes entertained that this process is slow and unsuitable for the provision of water in bulk. As Dr. Rideal points out, the necessity for such precautions is not avoided by the presence of a constant supply, for full pipes carrying a current of water will draw in surface drainage from the soil.

On the other hand, in the discussion of disinfection by heat—the method which is by far the most reliable and certain wherever it can be applied—Dr. Rideal states that he is indebted to an engineer for the principal portion of his chapter. The result is unsatisfactory. If anything is definitely known in practice, it is that hot air, whether moist or otherwise, requires an exposure of three hours at 284 deg. F. to yield absolute disinfection. Nevertheless, we are left with the hesitating statement that "no definite proof is given that moist air at about 230 deg. F. has absolutely germicidal properties if continued for two hours only." This tendency to hedge in the presence of well-ascertained facts is as deplorable as the tendency to generalise on facts imperfectly ascertained. Dr. Rideal speaks elsewhere of "the smallest really satisfactory hot air disinfecting chamber.' No hot air disinfecting chamber can be satisfactory, and the sooner this is definitely realised the better for the public.

* "Disinfection and Disinfectants (an Introduction to the Study of); together with an Account of the Chemical Substances used as Antiseptics and Preservatives." By Samuel Rideal, D.Sc. (London: Griffin, 1895.)

### A NEW PUBLIC LAVATORY FOR LONDON.

The St. Giles District Board of Works has set an excellent example to its brother boards in London in the very thorough manner in which it has carried out the work in connection with a new public lavatory for men and women just opened in New Oxford Street. This has been constructed beneath the roadway at the north end of Charing Cross Road, the men's department being entered from a refuge in the centre of the road, the women's from a staircase off the pathway. Both departments are well supplied and fitted. Hot and cold water is laid on to the lavatories, for the use of which and a clean towel 2d. is charged for each person.

The internal fittings are of the most modern description; the lavatories and passages are lined throughout with white tiles, and the water-closets are separated by marble partitions. Jennings' syphonic discharge apparatus, "The Closet of the Century," is the one used. Ventilation is effected by means of Blackman's air propeller, worked by water power, the waste water being used for flushing purposes; the ventilator is placed beneath a refuge formed in the middle of the carriage way at the north-west end of High Street, over which ornamental ventilating lamp columns have been erected, cast by Messrs. Pontifex and Co., Coleman Street, from designs prepared by Mr. George Wallace, C.E., engineer to the Board, who is responsible for the whole of the work. The contractors were Messrs. C. W. Killingback and Co., of Camden Town, while the sanitary arrangements were entrusted to Mr. George Jennings, of Palace Wharf, Lambeth. The roofs are of rolled steel girders and trough plates, supplied by Messrs. Westwood, Baillie, and Co., of Millwall, from which firm came also the staircases, made of iron framing fitted with Andrew's patent reversible treads.

The superficial area of the whole construction is 1,500 feet, and the depth from the level of the surface of the roadway 14 feet. The net cost of the work is stated to have been about £2,500.

The Board of Works for the St. Giles District is much to be congratulated on this provision for the convenience of the public, and much credit is also due to the Ladies' Sanitary Association, who have done a good work in insisting upon the need for such improved accommodation for women.

